# Spent Coffee Grounds Valorization as Bioactive Phenolic Source Acquired Antifungal, Anti-Mycotoxigenic, and Anti-Cytotoxic Activities

**DOI:** 10.3390/toxins14020109

**Published:** 2022-01-31

**Authors:** Ahmed Noah Badr, Marwa M. El-Attar, Hatem S. Ali, Manal F. Elkhadragy, Hany M. Yehia, Amr Farouk

**Affiliations:** 1 Food Toxicology and Contaminants Department, National Research Centre, Cairo 12622, Egypt; 2 Radioisotopes Department, Nuclear Research Center, Atomic Energy Authority, Cairo 11787, Egypt; m.elattar@agr.asu.edu.eg; 3 Food Technology Department, National Research Center, Cairo 12622, Egypt; 4 Department of Biology, College of Science, Princess Nourah bint Abdulrahman University, P.O. Box 84428, Riyadh 11671, Saudi Arabia; mfelkhadragy@pnu.edu.sa; 5 Food Science and Nutrition Department, College of Food and Agriculture Science, King Saud University, P.O. Box 2460, Riyadh 11451, Saudi Arabia; hanyehia@ksu.edu.sa; 6 Food Science and Nutrition Department, Faculty of Home Economics, Helwan University, Cairo 11221, Egypt; 7 Flavour and Aroma Chemistry Department, National Research Centre, Cairo 12622, Egypt; af.mansour@nrc.sci.eg

**Keywords:** antifungal, anti-mycotoxigenic, aflatoxins, cytotoxicity, degradation, detoxification, phenolic acids, flavonoids, spent coffee grounds, simulated media

## Abstract

Spent coffee grounds (SCGs), which constitute 75% of original coffee beans, represent an integral part of sustainability. Contamination by toxigenic fungi and their mycotoxins is a hazard that threatens food production. This investigation aimed to examine SCGs extract as antimycotic and anti-ochratoxigenic material. The SCGs were extracted in an eco-friendly way using isopropanol. Bioactive molecules of the extract were determined using the UPLC apparatus. The cytotoxicity on liver cancer cells (Hep-G2) showed moderate activity with selectivity compared with human healthy oral epithelial (OEC) cell lines but still lower than the positive control (Cisplatin). The antibacterial properties were examined against pathogenic strains, and the antifungal was examined against toxigenic fungi using two diffusion assays. Extract potency was investigated by two simulated models, a liquid medium and a food model. The results of the extract showed 15 phenolic acids and 8 flavonoids. Rosmarinic and syringic acids were the most abundant phenolic acids, while apigenin-7-glucoside, naringin, epicatechin, and catechin were the predominant flavonoids in the SCGs extract. The results reflected the degradation efficiency of the extract against the growth of *Aspergillus* strains. The SCGs recorded detoxification in liquid media for aflatoxins (AFs) and ochratoxin A (OCA). The incubation time of the extract within dough spiked with OCA was affected up to 2 h, where cooking was not affected. Therefore, SCGs in food products could be applied to reduce the mycotoxin contamination of raw materials to the acceptable regulated limits.

## 1. Introduction

Food by-products represent a critical global issue, where it is accumulated, representing global health and environmental issues. Food production increases annually due to the rise in the manufactured food demand, and therefore, the by-product quantities were raised. According to Ghosh et al. [1], the estimated population in 2100 will exceed 12 billion, which means the necessity toward sustainable food production, intelligent management, and exploitation of food and agro-waste. However, the rise in quantities of food by-products can be used as raw materials for the bio-economy [2]. Coffee is globally consumed due to its fantastic taste and compatibility with consumers′ preferences. During instant coffee processing, spent coffee grounds (SCGs) result as the main by-product obtained after steam extraction. The average weight of SCGs constitutes about 75% of the original coffee bean [3]. The global coffee production in 2020/2021 is about 10.17 million metric tons, which means about 6.5 million tons of SCGs have resulted from extraction [4]. The valorization of the SCGs through their implementation in biogas, food, feed, cosmetic, pharmaceutical, soil composites, and electricity production has been referred to before [5].

The SCGs showed concerning bioactive components such as insoluble dietary fiber, polysaccharides, lipids, amino acids, proteins, alkaloids, phenolics, minerals, and volatile compounds [6]. The previous investigations have focused on the SCGs’ bioactivity and their antioxidant [7], anti-inflammatory [8], dermatological, anti-melanogenesis [9], and metal scavenging properties [10]. The optimum conditions of solid/liquid SCGs-extraction using solvent systems to recover the antioxidant polyphenols were studied [11]. However, the eco-friendly solvent system that complies with the halal food market is steadily increasing and is required as a global trademark. Halal food laws are applied in several counties and communities, strictly prohibiting the use of haram (forbidden) animals or alcohol as a cause of intoxication [12].

The application of eco-friendly extraction may represent new characteristics for the gained extract. These characteristics could implement antibacterial, antifungal, and antiviral properties. Phenolic components can be found in a wide range of botanical meals and drinks (tea, coffee, and cocoa). The most prevalent phenolic molecules are coumarins, flavonoids, lignins, proanthocyanidins, and stilbenes [13,14]. The inactivation of enzyme synthesis and antibiofilm impact are two of the antifungal properties of phenolic molecules that have been widely reported [15,16]. Plants have antimutagens, antimicrobials, antioxidants, and anti-carcinogens to reduce mycotoxins′ toxicity and genotoxicity [17,18]. Antioxidants preserve cell membranes and biomolecules [19].

Moreover, phytochemicals cause cytotoxicity in fungi by altering cell membrane permeability and functionality, suppressing cytoplasmic and mitochondrial enzymes, suppressing enzymes associated with the formation of cell wall components, and changing the cell compartments, osmotic, and redox balance [20]. The previous investigation pointed to the phenolic content of the plant extract as an effective agent to suppress the fungal growth, which leads to an antifungal impact with its application [20]. Again, the phenolic content of the plant extract could regulate the fungal metabolism to decrease mycotoxin production. In this regard, the extract existing within fungal media may provide preservation for the applied product.

The present study evaluated the SCGs phenolics′ recovery utilizing an environmentally friendly, halal, and cost-effective extraction since isopropanol was applied under mild temperature conditions to preserve their activity. The antibacterial, antifungal, anti-mycotoxigenic, and cytotoxic activities of the SCGs isopropanol extract were evaluated to promote this by-product as a natural and inexpensive food preservative. The valorization of this by-product extract will recommend its application as a natural additive with safety properties.

## 2. Results

### 2.1. Characterization of SCGs Phenolic and Flavonoids

In this study, 15 phenolic acids and 8 flavonoids, in addition to caffeine (alkaloid), have been determined by UPLC in the SCGs isopropanol extract (Table 1). Rosmarinic and syringic acids were the most abundant phenolic acids in the isopropanol extract of the SCGs. In addition, the phenolic fractions of gallic, sinapic, and salicylic acids were recorded in the extract by valuable content. The phenolic acids of chlorogenic and caffeic manifested as nearby quantities in the isopropanol extract of the SCGs. Other important acids such as p-coumaric, ferulic, and vanillic have existed in small amounts. The SCGs extracted using isopropanol reflect a distinguished content of flavonoids, with the detection of eight compounds as shown in Table 1. Apigenin-7-glucoside, naringin, epicatechin, and catechin were significant in SCGs extract (Table 1). Notwithstanding, kaempferol, chrysin, quercetin, and rutin were detected in relatively low concentrations.

### 2.2. Cytotoxic Impact of SCGs Isopropanol Extract

The cytotoxic effects of the isopropanol extract on the viability of the HepG2 and OEC cell lines are presented in Table 2. Generally, the extract of SCGs showed some degree of cytotoxicity against the studied cells, reducing the cell viability percentage of the liver cancer cell line (HepG2) after treatment in comparison with healthy OEC cells, which indicated a selectivity of the studied extract, with the selectivity index (SI) 1.2 and 1.85 for the MTT 3-(4,5-Dimethylthiazol-2-yl)-2,5-Diphenyltetrazolium Bromide (MTT assay) (MTT) and the Sulforhodamine B colorimetric assay (SRB), respectively. The values obtained from the both assays of MTT and SRB assays were comparable. An SI value (<1) means the sample could be toxic and is not possible to be used as an herbal drug. Compared to the positive control, SCGs isopropanol extract was lower than Cisplatin′s reference drug (Table 2).

### 2.3. Isopropanol Bacterial (Diffusion Assay)

**Figure 1:**
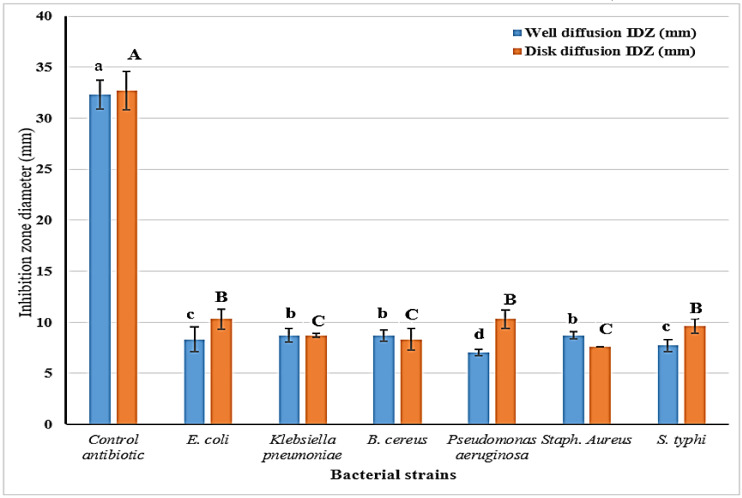
Antibacterial potency determined for the SCGs isopropanol extract against pathogenic strains of bacteria.

The SCGs’ antibacterial effect was recorded as having a valuable inhibition against tested strains of pathogenic bacteria (Figure 1). The inhibition zones, which were recorded by the two applied assays, were close to each other, emphasizing the efficient antibacterial effect of the extract versus used strains of food pathogens. According to the results, *E. coli* was the more sensitive strain influenced by the SCGs extract. It is worth mentioning that the SCGs inhibitory effect influenced both positive and negative strains of bacterial pathogens.

- The results are represented as means ± SEM, where (*n* = 3; (*p* ≤ 0.05); SEM: standard error means);
- IDZ: inhibition diameter of zone record. LSD: (0.577); letters are significantly different at *p* ≤ 0.05 level: capitals for disk diffusion and small for the well diffusion assays.

The data represented in the following figure reflect that the significant differences were recorded between the strains as in small letters (for each assay). No considerable differences show the results for the well diffusion antibacterial assay of *E. coli*, *Klebsiella*, Bacillus, and Staphylococcus. Again, the disk diffusion antibacterial assay of the same strains manifested no significant differences.

The inhibition zone diameter of the extract was recorded in nearby values, using two antibacterial assays, for the strains *Klebsiella* and *Bacillus*, as pathogenic bacteria. Compared to the inhibition impact using the standard antibiotic compound, the extract was recorded to have an inhibition influence against the investigated strains of pathogenic bacteria. It is worth mentioning that, for some bacterial strains, the results regarding the two assays were shown by significant differences. The finding could recommend applying the SCGs extract to be an antibacterial agent in the manufacturing of several products.

### 2.4. Isopropanol Extract Antifungal (Diffusion Assay)

**Figure 2:**
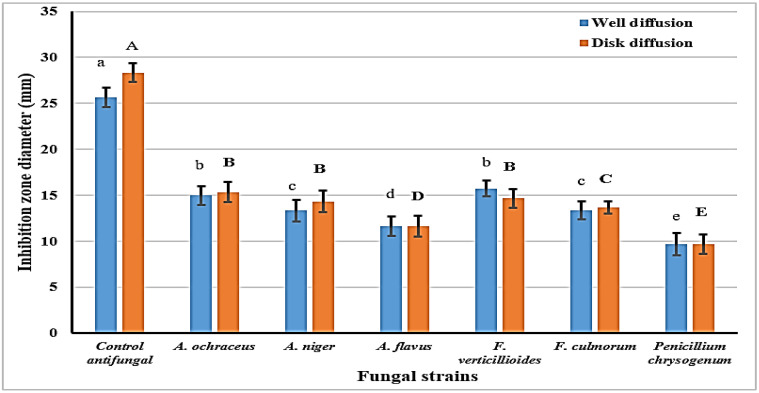
Antifungal impact determined for the SCGs isopropanol extract against toxigenic fungal strains using two assays.

Subsequently, the effect of the SCGs as an antifungal agent was evaluated against identified toxigenic strains of fungi known to produce mycotoxins. Tested strains mainly belonged to Aspergillus fungi, with two strains of Fusarium and Penicillium (Figure 2). The data represented the potency of the SCGs extract against the tested fungal strains, with a lower impact regarding the Penicillium strain. The extract′s influence on the strains is known, with capacity for ochratoxin production (*A. ochraceus*, *A. niger*). Regarding the evaluation of the SCGs extract, it was recorded with a considerable content of phenolic compound (Table 1); this could have participated in the manifested antifungal effect. It is clear that the antifungal effect, which is reflected here as inhibition zone diameter, was demonstrated by antifungal influence against the investigated strains close to 50% of the standard antifungal impact.

- Results are represented as means ± SEM, (*n* = 3; *p* ≤ 0.05; LSD-0.701; SEM: standard error means).
- Letters are significantly different at *p* ≤ 0.05 level: capitals for disk diffusion and small for the well diffusion assays.

### 2.5. Simulation Growth Inhibition for Fungal-Producing Toxin Strains on Liquid Media

**Figure 3:**
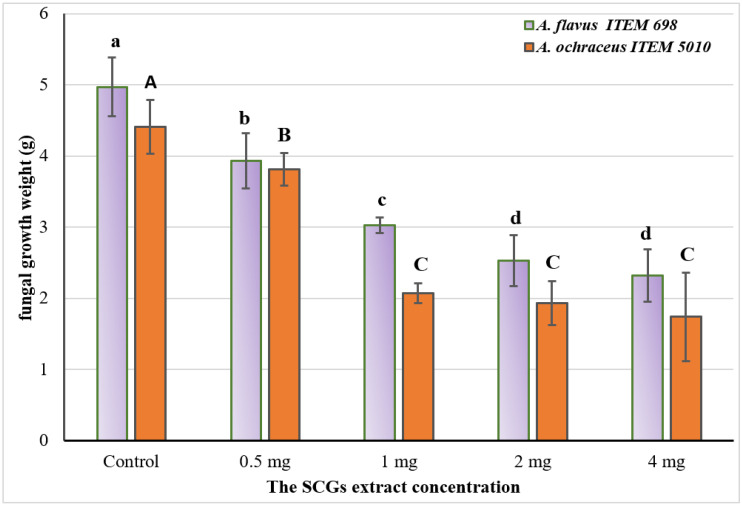
Reduction in fungal growth of two toxins producing *Aspergillus* strains using the SCGs extract.

To evaluate the reduction impact of the SCG to decrease the mycelial growth of two identified fungal-producing toxin strains, graduated concentrations of the SCG extract were applied in the liquid growth media of *A. flavus* and *A. ochraceus*. The results expressed in Figure 3 explain that the inhibition in the mycelia growth of fungi was raised by increasing the concentration of the extract.

- The results are represented as means ± SEM, where (*n* = 3; *p* ≤ 0.05); LSD = 0.397; SEM: standard error means).
- Values labeled with different letters are significantly different at *p* ≤ 0.05 level: capitals for *A. ochraceus* and smalls for *A. flavus* fungi. Upper and lower cases are for significant response of each strain to the treatment.

In this experiment, the authors aimed to simulate (using liquid media) the natural contamination that could happen by fungal strains known to have the capacity to produce mycotoxins. The positive impact of inhibition due to the SCGs extract indicates the extracts′ efficacy as antimycotic material. Generally, these results referred to 1 mg/mL as mediated concentration with efficacy inhibition. The influence of extract to reduce the mycelia′s fungal growth was significantly different between the investigated concentrations. It was reported that the values of the standard error mean at 1 mg of the SCGs extract tested were shown in low weights, which could indicate the effect’s stability. One more time, by the increment in extract concentration, the effect for the inhibition was raised, but the rate was lower for concentrations of more than 1 mg extract/mL media.

### 2.6. Simulation Degradation of Aflatoxins and Ochratoxin Production on Liquid Media

The data represented in Table 3 describe the increment in toxin production limitation by the increase in the SCGs extract concentrations applied in fungal media. Utilizing the previous media of *A. flavus* ITEM 698 and *A. ochraceus* ITEM 5010 strains growth, the quantities of aflatoxins (AFB_1_, AFB_2_, AFG_1_, and AFG_2_), as well as ochratoxin A (OCA), were determined. The results reflected that the greater the applied concentration of the SCGs extracts, the greater the toxin degradation ratio. However, more than 2 mL/mL of media concentration was mainly recorded with no significant differences.

The data in Table 3 indicate the degradation that may happen for the active groups of mycotoxin molecules due to the bioactivity of the SCGs extract. It was noticed that the degradation ratio, which has been recorded regarding the OCA by the increment in extract concentration in the growth media of fungi, was higher than that recorded regarding aflatoxins (AFs).

### 2.7. Application of the Spent on Brownies

Ready-made brownie powder was fortified using several ratios of spent coffee; hence, it was spiked by ochratoxin A, and mixes were evaluated for the incidence of toxin-content reduction. The results referred to incubation time and extract concentration in the dough as influential factors linked with ochratoxin reduction (Table 4). The removal of toxin amounts was observed by increasing the extract amount added to the dough ingredients. No significant difference was recorded for the OCA reduction in the dough after cooking. The reduction was significant at 3, 5, 7, and 10 mg of extract applied in the dough between 0 h and after 2 h of incubation before the dough was baked. The reduction was significant for the concentration of the SCGs extract (1 mg and 10 mg) applied in the dough.

## 3. Discussion

There are various undesirable properties, most notably the dose-related toxicity of chemical antifungals. Ideally, an antifungal should have null or reduced toxicity toward human cells [21]. Antifungal agents have reported drug resistance [22,23]. Therefore, it is necessary to discover new antifungal agents or safer alternatives to improve treatment against toxigenic fungi infection. In this regard, antifungal agents based on natural resources, such as phenolic compounds, are an alternative strategy to negate the rising antifungal drug resistance [24].

The phenolic compounds present in the SCGs are from the polyphenol-esters family, which mainly possesses bioactive functions including antioxidant, antibacterial, hepatoprotective, hypoglycemic, anticancer, and antiviral activities [6]. The bioactive molecules isolated from the SCGs have been reported to possess potential benefits as ingredients for the pharmaceutical, cosmetic, and food industries. For example, ethanolic extract can protect raw or cooked meat [25]. Chlorogenic and gallic acids were the most common phenolics in roasted coffee and SCGs, contributing to 80% of the total phenolics [8,26]. The yield and kind of bioactive components recovered from the SCGs are defined according to coffee species, extraction method, storage conditions, and instant-coffee manufacturing efficiency. Increased coffee roasting intensity (from medium to dark) reduced the phenolic acids of chlorogenic (6%), gallic (15%), p-coumaric (33%), ferulic (14%), quercetin (14%), and catechin (60%) due to isomerization. At the same time, this treatment increased ellagic acid (50%) without affecting caffeic acid and rutin contents [8].

The present extract was richer in flavonoid compounds than the previous investigations of different extraction systems. Four flavonoids have been identified in the SCGs using ethanol and microwave extraction techniques: epicatechin, catechin, rutin, and quercetin [8]. However, in the present extract, there were eight flavonoids recorded. Flavonoids own several health functions and elicited protective effects, including anti-inflammatory, antioxidant, antiviral, and anti-carcinogenic effects. Recently, apigenin has spurred interest due to its low intrinsic toxicity and antidiabetic and anti-inflammatory activities. Apigenin-7-glucoside showed higher antifungal activity on *Candida* and cytotoxic effect on colon cancer cells than apigenin [27]. In turn, naringenin has been reported to exhibit antioxidant activity [28].

Caffeine is one of the essential bioactive components found in SCGs in a remarkable concentration (Table 1), consistent with Kovalcik et al. [6]. It can exert many biological activities such as the possible link to depression by reducing pro-inflammatory mediators tumor necrosis factor alpha (TNF-α), Interleukin- 1β (IL-1β), Interleukin-6 (IL-6), and interferon-c (IFNc), probably via the Jun N-terminal kinase (JNK1/2) and p 38 mitogen-activated protein kinases (p 38 MAPK) signaling pathway [29]. Some antifungal agents have been drug-resistant [22,23]. New antifungal compounds or better alternatives should be discovered to increase treatment efficacy against toxigenic fungus infection. In this regard, antifungal agents derived from natural resources, such as phenolic compounds, provide an alternative strategy for addressing developing antifungal drug tolerance [24]. Many of these compounds possess antimicrobial effects [30,31]. The mechanism of phenolic compounds to inhibit microbial contamination is linked mainly to the antioxidant potency possessed by the phenolic extract [32].

Concerning the results, the SCG possessed better antifungal potency, making it qualified to decrease the contamination by toxigenic fungi where it exists (Figure 2 and Figure 3). Moreover, the toxins which these strains of fungi could secrete were recorded limited or degraded if the extract was present in their growth media (Table 3). The mycotoxin reduction compared to the control displayed the interaction of the SCGs extract’s constituents with the growth factors of toxigenic fungi. These developments, which occurred in the media content through the extracts′ existence, lead to toxin secretion reduction [33]. The AFs are a family of health-hazard chemicals generated from polypeptides [34]. More than 20 genes grouped in the DNA sequence area must be synthesized. As transcription agents, the genes encode various enzymes involved in toxin production [35]. The mechanism by which the reduction happened to aflatoxins secretion proposed a link to gene docking between genes of aflatoxin production in the fungal cell and polyphenol compounds.

Several prior studies have looked at the effect of plant phytochemicals on toxigenic fungi′s aflatoxin gene cluster and the aflatoxin biosynthesis pathway. Phytochemicals such as phenolic acids are also known to inhibit gene expression in the aflatoxin pathway [18,36]. Furthermore, plant phytochemicals play a key role in toxigenic fungus gene regulation [20,37]. In the same ways, and where Ochratoxin A is produced by another *Aspergillus* fungal strain, the mechanism of ochratoxin reduction in media could be linked to the extract’s contents of bioactive phenols. This suggestion led the authors to investigate the extract efficacy even when the food products are contaminated by ochratoxin. Forward of this, the application of the SCGs extract in food products containing spiked ochratoxin resulted in safer properties, where the extract recorded a reduction impact in synthetic media (Table 4). The spiked-ochratoxin reduction in a simulated food model shows the extract′s effectiveness as a safety agent that could be processed for the safety properties of the final product. The reduction recorded for toxin production in media, which was noticed for the results in Table 3, could be elucidated through the phytochemical impact on the toxin gene regulation [38].

In agreement with our results, Gigliobianco et al. [39], found that the cytotoxicity of aqueous SCGs extract at 80 °C in keratinocyte HaCaT cells was ineffective on cell viability at concentrations lower than 3 mg/mL. Coffee and its spent grounds contain many bioactive constituents that affect the human body, such as caffeine, caffeic acid, chlorogenic acids, trigonelline, diterpenes, and melanoidins. Some have demonstrated potential anti-carcinogenic effects in animal models and human cell cultures and may play a protective role against many types of cancer such as colorectal cancer [40].

## 4. Conclusions

The current study encourages researchers to exploit waste from food, such as SCGs, which are commonly trashed, to produce extracts with added values. Concerning the promising bioactive phenolics and flavonoids components obtained, the anti-mycotoxigenic impact and the cytotoxicity of SCGs isopropanol extract were evaluated during the current study, along with a simulation experiment for ochratoxin reduction in brownies. Phenolic compounds have well-known bioactivity against oxidative stress caused by mycotoxin contamination, in addition to the caffeine, which positively reduced ochratoxin reduction. In agreement with the above facts, the extract was shown by an antifungal effect applied in diffusion assays against toxigenic fungal strains, especially *A. flavus* and *A. ochraceus*. The extract is more efficient for the anti-*Fusarium* followed by the anti-ochraceus fungi activity. Therefore, SCGs could be applied in food products to reduce the mycotoxin contamination of raw materials to the acceptable regulated limits. Additionally, the examined extract′s moderate cytotoxic and antibacterial activities represent a business opportunity to have nutraceutical foods with healthy functions.

## 5. Materials and Methods

### 5.1. Materials, Chemicals, and Microorganisms

Spent coffee was gifted to the research team as a free sample from Misr Coffee (10th. of Ramadan Ind. City, Cairo, Egypt Industrial Company). The powder was milled to a close micronize granules (40 mesh) for the extraction and application steps. The powder was immediately dried (38 ± 2 °C) using a Hot-air oven (Model ED 56, Binder GmbH, 78532 Tuttlingen, Germany) until it was completely dried.

Six strains of bacteria including *Staphylococcus aureus* ATCC 33591, *Bacillus cereus* ATCC 11778, *Klebsiellapneumoniae* ATCC 27736, *Escherichia coli* ATCC 11229, *Pseudomonas aeruginosa* ATCC 9721, and *Salmonella typhi* ATCC 14028 were applied for the antibacterial assay. Antifungal assay was determined against the fungi strains of *A. flavus* item 698, *A. ochraceus* item 7043, *A. niger* item 9568, *Penicillium chrysogenum* ATCC 10106, *F. culmorum* KF 846, and *F. verticillioides* FM 19.

### 5.2. Preparation of Spent Coffee Extract

Isopropanol extraction assay was applied to gain the SCGs bioactive components following the methodology described by Abdel-Salam et al. [41], with modifications. A contentious system using a peristaltic pump (Model S300-12B, Baoding Ditron Electronic Technology Co., Baoding, China) with a filter, connected to a horizontal-double jacket basin containing the powder of spent coffee submerged in aqueous isopropanol (80%) at 40 °C/4 h was used. The gained extract was collected after the end process, concentrated to known volume using a rotary evaporator (Heidolph, HeiVAP, GmbH, Landsberger, Germany), and was then was kept in an amber bottle for further analyses and applications.

### 5.3. Determination of Phenolic Acids and Flavonoids

The analysis was performed using an Acquity H class UPLC system equipped with a Waters Acquity PDA detector (Waters, Milford, MD, USA). The condition and the column characteristics were the same as described by Stuper-Szablewska et al. [42]. As the eluent for the chromatographic separation, a solution of acetonitrile: 2% acetic acid in water (pH = 2) was utilized (gradient). External standards were used to detect the concentrations of phenolic acids at = 320 and 280 nm, and the detection limit was 1 μg. It was possible to determine the existence of certain substances by matching the retention times of examined peaks to those of standard references and then conducting further analyses using a smaller volume of the standard added with the estimated samples.

### 5.4. Determination of Antibacterial and Antifungal Effects

The antimicrobial activity of the spent coffee extract was determined by measuring the diffusion assays according to the methodology of Abdel Razek et al. [43]. Applied strains of bacteria were reactivated from lyophilized stocks on Tryptic soy broth media. The activated strains were spread on Tryptic soy agar plates, the disks or wells were loaded using 100 µL of spent extract. The impact of applied extract for the inhibition was recorded as clear zone diameter (mm) for each strain: the greater the inhibition zone diameter, the more effective the concentration. The antifungal effect was determined against the fungal strains cultured on Czapek-dox agar media, where the disk and well diffusion assays were applied using isopropanol SCGs extract.

### 5.5. Determination of SCGs Cytotoxic Effect Using the Tetrazolium-Based (MTT) Assay

Human liver cancer (HepG2) and human healthy oral epithelial (OEC) cell lines were cultured at a density of 1 × 10^4^ cells/well (100 µL) in a culture medium of the Dulbecco’s Modified Eagle Medium (DMEM) supplemented with antibiotics (10,000 U of penicillin and 10 mg of streptomycin in 0.9% saline) and 10% serum of the phosphate buffer saline (PBS). They were then incubated for 24 h at 37 °C and 5% CO_2_. After attachment for 24 h, a serially diluted extract was applied at concentrations ranging from 1000 to 0.01 µg/mL for OEC and from 200 to 0.01 µg/mL for HepG2 cells. As parallel, a positive control (Cisplatin) at concentrations ranging from 400 to 0.01 µg/mL was applied. Subsequently, 10 µL of a 12-mM MTT stock solution (5 mg/mL MTT in sterile PBS) was added to each well. After incubation for 4 h at 37 °C, the MTT solution was eliminated, and the precipitated purple formazan crystal was dissolved in dimethyl-sulphoxide (DMSO) for 20 min. Negative control of 10 µL of the MTT stock solution was added to 100 µL of an uncultured medium. The absorbance was measured at 540 nm using a BMG LABTECH^®^- FLUOstar Omega microplate reader (Ortenberg, Germany). The absorbance was measured at 570 nm.

**[(OD _sample_ − OD _blank_)/(OD _control_ − OD _blank_) × 100%]** (1)

where; OD _sample_: optical density of the sample.
OD _blank_: optical density of the blank (DMSO).
OD _control_: optical density of the control.

The curve was illustrated based on the variation of the proportions of surviving cells according to concentrations, and IC_50_ was calculated using a sigmoidal curve obtained [44].

### 5.6. Determination of the SCGs Cytotoxic Effect Using Sulforhodamine B (SRB) Assay

The cytotoxic activity of the extract was assessed again by SRB assay against HepG2 and OEC cell lines. Aliquots of 100 μL cell suspension (5 × 10^3^ cells) were placed in 96-well plates and incubated in complete media for 24 h. Cells were treated with another aliquot of 100 μL media containing the extract or the positive control (Cisplatin) at various concentrations ranging from 0.01–1000 µg/mL. After 72 h of drug exposure, cells were fixed by replacing media with 150 μL of 10% TCA and incubated at 4 °C for 1 h. The trichloroacetic acid solution (TCA) solution was removed, and the cells were washed five times with distilled water. Aliquots of 70 μL SRB solution (0.4% *w*/*v*) were added and incubated in a dark place at room temperature for 10 min. Plates were washed three times with 1% acetic acid and air-dried overnight. Then, 150 μL of 10 mM trisaminomethane (TRIS) was added to dissolve the protein-bound SRB stain; the absorbance was measured at 540 nm using a BMG LABTECH^®^- FLUOstar Omega microplate reader (Ortenberg, Germany) [45].

### 5.7. Selectivity Index (SI)

Selectivity index (SI) is a ratio of the toxic concentration of a sample against its effective bioactive concentration. The higher ratio of SI represents a more effective and safer drug when used during in vivo treatment [46]. The following equation evaluated the SI value:

**SI = IC_50_^No cancer cell^/IC_50_^Cancer cell^** (2)

### 5.8. Simulated Experiment to Evaluate the Anti-Mycotoxigenic Impact

The influence of the SCGs extract against toxigenic fungal strains (*A. flavus* ITEM 698 and *A. ochraceus* ITEM 7043) was determined. The expected inhibition for fungal growth and toxin reduction of these known strain-producing fungi was evaluated at several concentrations (0.5, 1, 1.5, 2 mg extract/100 mL media). The 10^5^ spore/mL concentration was suspended in a 1 L conical flask containing 250 mL yeast extract sucrose (YES). The flasks of control (SCGs-free) and treatments were incubated (5 days/22 °C for growth change evaluations and 12 days/28 °C for toxin production changes). The experiment was performed in triplicate and statistically evaluated.

### 5.9. Application of Spent Coffee in Brownies for Ochratoxin A Evaluation

A ready-made powder for cookies was purchased for preparing a food model. Such a model was utilized for evaluating spiked-OCA reduction that linked to SCGs-fortification. Five ratios were designed to assess the fortifications impact as 1, 3, 5, 7, and 10% of the SCGs added to the recipe. The spiked cookies (+ve control), the control (-ve control), and fortified samples (treatments) were evaluated at (0, 2 h, and 4 h) of dough incubation and after cooking. The OCA reduction is directly proportional to the SCGs’ effective ratio of fortification. The samples were taken in triplicate and analyzed statistically.

### 5.10. Mycotoxin Determination

High-performance liquid chromatography, Agilent 1100 (Agilent Technologies, Hewlett-Packard Strasse 876,337 Waldbronn, Germany), was utilized for the AFs and OCA determination. The AFs mobile phase was water: acetonitrile: methanol (6:3:1). The chromatographic separation was performed with an Extend-C18, Zorbax column (250 mm × 5 mm; 46 µm, Agilent Co., Santa Clara, CA, USA). The column temperature was 40 °C, and the flow rate was 1.0 mL/min; the injection volume was 20 µL for samples and standard. The detector was adjusted at 360/440 nm for the excitation and the emission wavelength, respectively. Data were integrated and recorded using a Chem-Station software Manager Hewlett-Packard (Agilent Co., Santa Clara, CA, USA). The OCA was extracted and evaluated from the media, similar to the methodology of Badr et al. [47]. The OCA mobile phase was a mixture of acetonitrile: water: methanol (46:46:8, *v*/*v*), and the flow rate was 1.0 mL/min. The OCA quantification was performed by comparing the retention time against the standard. The OCA identity was confirmed at 274 and 440 nm for excitation and emission wavelengths, respectively, compared to the OCA peak of the standard. The injection volume was 20 µL, and the retention time was approximately 11 min. The analysis has a detection limit of 0.01 µg/g. A computing integrator (Millenium-32v., 3.05) was utilized to compare the peak area with the proper standard curve, which resulted in an accurate measurement.

### 5.11. Statistical Analysis

The results were expressed as means ± standard deviation (SD) of at least three replicates. The analysis of variance (ANOVA) was used to assess the significant difference between the mean values, and Duncan′s multiple range test was calculated (*p* = 0.05). The statistical data analyses were performed using Graph Pad Prism 7 (Graph Pad Software Inc., San Diego, CA, USA).

## Figures and Tables

**Table 1:** Contents of phenolic acids, flavonoids, and caffeine determined in SCGs isopropanol extract.

**Phenolic Acid Contents of the SCGs Extract**			
**Compound**	**Quantities** **(µg/g)**	**Compound**	**Quantities** **(µg/g)**
Gallic acid	18.15 ± 0.63	Sinapic acid	17.05 ± 0.54
Protocatechuic acid	0.82 ± 0.04	(S)-(-)-rosmarinic acid	0.92 ± 0.02
p-Hydroxybenzoic acid	3.65 ± 0.09	Ferulic acid	3.64 ± 0.17
Gentisic acid	8.6 ± 0.13	Salicylic acid	12.67 ± 0.27
Chlorogenic acid	7.38 ± 0.31	p-coumaric acid	0.19 ± 0.03
Caffeic acid	7.2 ± 0.73	Cinnamic acid	0.495 ± 0.06
Syringic acid	64.14 ± 0.83	(R)-(+)-rosmarinic acid	176.43 ± 1.27
Vanillic acid	0.47 ± 0.06	-	-
**Flavonoid Contents of the SCGs Extract**			
**Compound**	**Quantities** **(µg/g)**	**Compound**	**Quantities** **(µg/g)**
Catechin	16.86 ± 0.27	Quercetin	1.38 ± 0.05
Epicatechin	53.83 ± 1.02	Apigenin-7-glucoside	1717.04 ± 3.54
Rutin	4.87 ± 0.11	Kaempferol	3.18 ± 0.41
Naringin	74.49 ± 0.69	Chrysin	1.34 ± 0.18
**Alkaloid Contents of the SCGs Extract**			
**Compound**		**(µg/g)**	
Caffeine		208.93 ± 2.05	

The data were expressed as means ±SEM (where *n* = 3, LSD = 0.704, *p* ≤ 0.05); SEM: standard error means; LSD: least significant differences;.SCGs: spent coffee grounds,.

**Table 2:** Cytotoxic activity of SCGs isopropanol extract against HepG2 and OEC cell lines using MTT and SRB assays.

Extract	Cell Lines	IC_50_ (μg/mL)	SI
Cisplatin	HepG2	66.69	1.3
	OEC	87.69	-
Isoprpanol extract (MTT)	HepG2	112	1.2
	OEC	133.7	-
Isopropanol extract (SRB)	HepG2	94.03	1.85
	OEC	174.1	-

For the MTT test: the value of the LSD was (4.542); R^2^ = 0.9912. For the SRB test: the value of the LSD was (3.139); R^2^ = 0.995; IC_50_: the half-maximal inhibitory concentratio; SI: selectivity index.

**Table 3:** Reduction in aflatoxins and Ochratoxin A produced by *Aspergillus* toxin-producing strains using the graduated concentrations of the SCGs extract.

Concentration	AFB_1_	AFB_2_	AFG_1_	AFG_2_	OCA
Control	258.0 ±1 4.55 ^a^	184.0 ± 15.17 ^a^	208.3 ± 14.82 ^a^	175.7 ± 12.37 ^a^	910.7 ± 16.77 ^a^
0.5	194.3 ± 13.09 ^b^	138.3 ± 13.81 ^b^	174.3 ± 14.26 ^b^	148.0 ± 12.19 ^b^	612.6 ± 17.05 ^b^
1 mg	141.4 ± 13.14 ^c^	103.7 ± 13.69 ^c^	118.3 ± 13.37 ^c^	94.6 ± 10.57 ^c^	257.7 ± 15.22 ^c^
2 mg	132.0 ± 12.88 ^cd^	99.0 ± 11.74 ^cd^	103.6 ± 12.07 ^d^	86.3 ± 11.77 ^cd^	226.8 ± 14.14 ^d^
4 mg	119.3 ± 16.23 ^d^	88.3 ± 11.81 ^d^	98.7 ± 12.54 ^d^	79.0 ± 11.41 ^d^	201.0 ± 13.79 ^e^

The results are represented as means ± SD, where (*n* = 3; *p* ≤ 0.05); SD: Standard Deviation). AFB_1_: aflatoxin B_1_; AFB_2_: aflatoxin B_2_; AFG_1_: aflatoxin G_1_; AFG_2_: aflatoxin G_2_; OCA: ochratoxin A. The values represented by different superscripted letters are significantly different for the same coulumn.

**Table 4:** Spiked Ochratoxin A reduction in a simulated model of contaminated brownies fortified by the SCGs extract.

	Control	1 mg *	3 mg *	5 mg *	7 mg *	10 mg *
Zero-time	850 ± 13.14 ^a^	837 ± 18.54 ^a^	817 ± 17.63 ^a^	811 ± 19.05 ^a^	796 ± 17.49 ^a^	779 ± 15.17 ^a^
2 h incubation	852 ± 14.56 ^a^	742 ± 13.79 ^b^	711 ± 19.13 ^b^	671 ± 18.21 ^b^	524 ± 17.71 ^b^	319 ± 27.64 ^b^
4 h incubation	851 ± 12.81 ^a^	737 ± 11.27 ^b^	691 ± 16.23 ^b^	588 ± 28.34 ^c^	502 ± 26.25 ^b^	307 ± 37.57 ^b^
After cooking	836 ± 17.37 ^a^	731 ± 10.91 ^b^	689 ± 15.84 ^b^	574 ± 27.77 ^c^	495 ± 26.84 ^b^	282 ± 33.93 ^b^

The results are represented as means ± SD, where (*n* = 3; *p* ≤ 0.05; SD: Standard Deviation). The values represented by different superscripted letters are significantly different. (*) refers to the concentration of spent extract applied in the dough.

## Data Availability

All the data regarding this work were represented inside this manuscript.
